# Combination Treatment of C16 Peptide and Angiopoietin-1 Alleviates Neuromyelitis Optica in an Experimental Model

**DOI:** 10.1155/2018/4187347

**Published:** 2018-02-18

**Authors:** Yuanyuan Zhang, Kewei Tian, Hong Jiang, Beibei Wang, Shu Han

**Affiliations:** ^1^Institute of Anatomy and Cell Biology, Medical College, Zhejiang University, Hangzhou, China; ^2^Department of Electrophysiology, Sir Run Run Shaw Hospital, Medical College, Zhejiang University, Hangzhou, China; ^3^Core Facilities, Zhejiang University School of Medicine, Hangzhou, China

## Abstract

Neuromyelitis optica (NMO) is an autoimmune inflammatory demyelinating disease that mainly affects the spinal cord and optic nerve, causing blindness and paralysis in some individuals. Moreover, NMO may cause secondary complement-dependent cytotoxicity (CDC), leading to oligodendrocyte and neuronal damage. In this study, a rodent NMO model, showing typical NMO pathogenesis, was induced with NMO-IgG from patient serum and human complement. We then tested whether the combination of C16, an *α*v*β*3 integrin-binding peptide, and angiopoietin-1 (Ang1), a member of the endothelial growth factor family, could alleviate NMO in the model. Our results demonstrated that this combination therapy significantly decreased disease severity, inflammatory cell infiltration, secondary demyelination, and axonal loss, thus reducing neural death. In conclusion, our study suggests a possible treatment that can relieve progressive blindness and paralysis in an animal model of NMO through improvement of the inflammatory milieu.

## 1. Introduction

Neuromyelitis optica (NMO) is an autoimmune inflammatory disease that selectively targets the optic nerves and spinal cord, leading to blindness and paralysis [1]. Since NMO patients often present demyelinating lesions in the central nervous system (CNS), it has long been considered a variant of multiple sclerosis (MS); however, recent data suggest that its pathogenesis may be different [2]. The immunopathology of NMO includes restricted demyelination and inflammation of the optic nerves and several spinal segments [3]. The unique pathogenesis that distinguishes NMO from MS is that NMO-IgG is present in most NMO patients. NMO-IgG selectively binds to the water channel aquaporin-4 (AQP4), which is densely expressed in astrocytic foot processes at the blood-brain barrier (BBB), thus destroying the integrity of the BBB. Pathological characteristics of NMO include loss of AQP4 and glial fibrillary acidic protein (GFAP) and granulocyte and macrophage infiltration, as well as demyelination and axonal injury mainly in the spinal cord and optic nerves of NMO patients [4]. Therefore, the pathogenicity of NMO-IgG (anti-AQP4 autoantibodies) and human complement has been investigated, contributing to the initial NMO model [4–8].

Worldwide, the prevalence of NMO is far lower than that of MS, but NMO is more common in Asian populations [3]. NMO has a worse outcome than MS, with frequent and early relapses [3]. It is crucial to distinguish between NMO and MS because some MS treatments exacerbate NMO [9]. Currently, treatment for NMO aims to control the inflammatory damage in acute attacks and to manage the disease properly to avoid relapses [10]. The former is achieved using high-dose intravenous corticosteroids, and the latter by low-dose corticosteroids and immunosuppressants, including azathioprine, rituximab, methotrexate, mycophenolate mofetil, and mitoxantrone [11]. However, the long-term application of these drugs causes apparent side effects. Other treatments, such as plasmapheresis or plasma exchange, are expensive if used for long periods of time.

The synthetic C16 peptide (KAFDITYVRLKF), representing a functional laminin domain, selectively binds to the integrin *α*v*β*3, interfering with leukocyte transmigration across the endothelial cell layer *in vitro* [12, 13]. Previous studies also have revealed that the presence of the integrin *α*v*β*3 could decrease monocyte binding to intercellular adhesion molecule-1 (ICAM-1) and block monocyte migration across the endothelium [13]. It also alleviates monocyte extravasation and macrophage activation in spinal cord contusion models *in vivo* [13]. Angiopoietin-1 (Ang1) protein is a member of the novel angiopoietin growth factor family that binds to the receptor tyrosine kinase Tie2 and regulates several aspects of the angiogenic process, which is important for stabilizing the endothelium and reducing endothelial permeability [13]. Our previous studies have revealed that the combination treatment of C16 and Ang1 could improve the inflammatory milieu and delay the onset of motor symptoms in an experimental autoimmune encephalomyelitis animal model, which is frequently used as a model for MS [14–16]. In the present study, we tested the effects of this combination treatment on a novel rodent NMO model, which has clinical characteristics of severe inflammation and demyelination in the CNS. Multiple molecular, histological, and immunohistochemical assays in addition to behavioral and electrophysiological tests were adopted to assess inflammation, axonal loss, demyelination, and neuronal apoptosis in the retina, optic nerve, and spinal cord as well as the progression of NMO.

## 2. Materials and Methods

### 2.1. Ethics Statement

The study protocols were approved by the Ethics Committee of Zhejiang University. All animal procedures used in this study were carried out in accordance with the National Institute of Health Guide for the Care and Use of Laboratory Animals.

### 2.2. Induction and Treatment of NMO in Rats

Ninety Lewis rats (female, 10- to 12-week-old, and 200–250 g) were used in this study. To induce NMO, serum was obtained from two patients with an established diagnosis of NMO and strong AQP4 autoantibody serum positivity. Human NMO immunoglobulin G was purified as described previously [17]. AQP4-Ab titers were independently measured by fluoroimmunoprecipitation and cell-based assays [5].

The rats were anesthetized by 1% Nembutal (40 mg/kg, ip). For intraventricular injection, the coordinates of the injections were as follows: anteroposterior (AP) −0.7 mm; mediolateral (ML) −1.7 mm from the bregma; and depth, 5 mm from the skull surface [8]. An osmotic minipump (Alzet 1003D, USA) with a catheter was used to deliver 10 *μ*g NMO-IgG and 50 *μ*L (5 *μ*g/*μ*L) human complement (Complement Technology, Tyler, TX, USA) for 3 days (1 *μ*L/hr). Meanwhile, the vertebrae were carefully separated using two fine tweezers to expose the lumbar spinal cord (L4–L5); the same amount of NMO-IgG and human complement was infused for 3 days intrathecally with Alzet 1003D minipumps and catheters.

NMO rats were randomized into two groups: vehicle-treated (*n* = 30) and C16 + Ang1- (C + A-) treated (*n* = 30). Non-NMO animals (*n* = 30), receiving normal serum with human complement, were also included as a control group.

The C16 peptide (KAFDITYVRLKF, Shanghai Science Peptide Biological Technology Co. Ltd., China) solution (4 mg/mL) was prepared as described previously [13, 14]. Briefly, the peptide was dissolved in distilled water with 0.3% acetic acid, sterilized through a 0.22 *μ*m disc filter, and neutralized to pH 7.4 with NaOH.

Ang1 protein (KLENYIVENMKSEMAQIQQNAVQNHTATMLEIGTSLLSQT AEQTRKLTDVETQVLNQTSRLEIQLLENSLSTYKLEKQLLQQTNEILKI, Shanghai Science Peptide Biological Technology Co. Ltd., China) was dissolved in distilled water to a final concentration of 800 *μ*g/mL. The mixture of 0.5 mL of Ang1 (400 *μ*g) and 0.5 mL of C16 (2 mg) was injected intravenously via the tail vein every day for 1 week. The first dose was given immediately after NMO induction. Because scrambled C16 acts as an *α*v*β*3 and *α*5*β*1 antagonist, reverse C16 has some binding activities for endothelial cells in culture [13], and the Ang1-derived peptide QHREDGS can promote cell survival and adhesion [18, 19], we could not use C16- and Ang1-based control peptides as controls. Thus, phosphate-buffered saline (PBS) was injected as a vehicle.

### 2.3. Animal Scoring and Neurophysiological Testing

Disease severity was assessed on a daily basis by two observers using a scale ranging from 0 to 10: 0, normal; 1, reduced tone of the tail; 2, limp tail, impaired righting; 3, absent righting; 4, gait ataxia; 5, mild paraparesis of the hindlimb; 6, moderate paraparesis; 7, severe paraparesis or paraplegia; 8, tetraparesis; 9, moribund; and 10, death [7].

Cortical somatosensory-evoked potentials and cortical motor-evoked potentials were recorded at 1 and 8 weeks postinjection (pi), as described previously [12, 14–16].

### 2.4. Perfusion and Tissue Processing

To prepare cryosections, rats were anesthetized with sodium pentobarbital and transcardially perfused with 4% paraformaldehyde at 3 days, 1 week, and 8 weeks (*n* = 10/group at each time point) after NMO-IgG injection. The spinal cords, brains, and eyes, with the optic nerve cut at 1 mm behind the eye globe, were collected. The tissues were fixed in 4% paraformaldehyde overnight at 4°C and were sequentially cryoprotected in 15% and 30% sucrose in PBS, embedded in Tissue-Tek optimal cutting temperature compound (Sakura Finetechnical, Tokyo, Japan), and made into cryosections (20 *μ*m thickness). The sections were then mounted on 0.02% poly-L-lysine-coated slides. The remains of the CNS tissue were fixed in 5% glutaraldehyde solution and examined by transmission electron microscopy.

### 2.5. Histology

The sections were stained with hematoxylin and eosin to observe infiltration of immune cells, and the severity of cell infiltration was scaled using a 5-point scale system: 0, no inflammation; 1, cellular infiltration only around the blood vessel and meninges; 2, mild cellular infiltration in the parenchyma (1–10/section); 3, moderate cellular infiltration in the parenchyma (11–100/section); and 4, serious cellular infiltration in the parenchyma (100/section) [12, 20].

The sections were stained with cresyl violet (Nissl staining) to observe the neuronal structure, as described previously [12, 20]. Neuronal counts were made for large, multipolar spinal cord anterior horn motor neurons and the pyramid-shaped motoneurons of the precentral gyrus, and they were restricted to cells with a well-defined nucleus and a cell body that displayed adequate amounts of the endoplasmic reticulum.

Demyelination was evaluated by Luxol fast blue staining and scored using a 6-point scale system: 0, normal white matter; 1, rare foci; 2, a few areas of demyelination; 3, confluent perivascular or subpial demyelination; 4, massive perivascular and subpial demyelination involving one half of the spinal cord with the presence of cellular infiltration in the CNS parenchyma; and 5, extensive perivascular and subpial demyelination involving the whole cord section with the presence of cellular infiltration in the CNS parenchyma [12, 20].

Axonal loss was estimated by Bielschowsky silver staining using the following 4-point scale: 0, no axonal loss; 1, a few foci of superficial axonal loss involving less than 25% of the tissues; 2, foci of deep axonal loss that encompassed over 25% of the tissue; and 3, diffused and widespread axonal loss [12, 20].

Five transverse sections from each animal were selected at random, and digital photomicrographs were obtained at 200x magnification in three visual fields per section. Quantification of the histological results was done by investigators blinded to the treatments.

### 2.6. Immunohistochemistry

The sections were preincubated with PBS containing 0.1% Triton X-100 (PBST) for 10 min at room temperature, then blocked with 10% normal goat serum in PBST (blocking solution) for 1 h, followed by incubation in blocking solution overnight at 4°C with one of the following antibodies: mouse anti-neurofilament M (NF-M, 1 : 500; United States Biological, Salem, MA, USA), glial fibrillary acidic protein (GFAP, 1 : 200; MAB360, Millipore), myelin basic protein (MBP, 1 : 100; NE1018, Sigma), and aquaporin-4 (AQP4, 1 : 200; SC20812; Santa Cruz Biotechnology), followed by the appropriate fluorescent secondary antibody (1 : 200, Invitrogen, Grand Island, NY, USA). The sections were mounted with Antifade Gel/Mount aqueous mounting media (SouthernBiotech, Birmingham, AL, USA). All control sections were incubated in PBS without primary antibodies. Five transverse sections from each animal were randomly selected, and digital photomicrographs were obtained at 200x magnification in three visual fields per section. Areas that exhibited immunoreactivity were analyzed with NIH ImageJ software.

Rabbit anti-activated caspase-3 (1 : 500; Cayman Chemical, Ann Arbor, MI, USA) and CD45 (1 : 100, Santa Cruz Biotechnology) were incubated overnight at 4°C. Sections were incubated with secondary biotinylated goat anti-rabbit IgG antibody (1 : 400; Vector Laboratories, CA) for 1 h at 37°C followed by avidin-biotin peroxidase complex (ABC kit, Thermo Fisher Scientific, MA). After incubation for 5 min with 0.02% 3,3′-diaminobenzidine and 0.003% H_2_O_2_ in 0.005 Tris-HCl, the sections were counterstained with hematoxylin. Controls without adding primary antibody were used to further confirm the specificity of the immunohistochemical labeling. Five transverse sections from each animal were selected at random, and images were photographed under 200x magnification in three visual fields per section. Caspase-3-immunoreactive cells were counted on sections of the retina as well as anterior horns of each spinal cord.

### 2.7. Counting of Retinal Ganglion Cells (RGCs)

RGCs were retrospectively labeled by bilateral stereotactic injection of FluoroGold into the superior colliculus 5 days before treatment with the NMO-IgG and human complement (*n* = 3/group). Briefly, the brain surface was exposed by drilling the parietal bone using a stereotactic apparatus. A total of 2.1 mL of 5% FluoroGold (Fluorochrome, Denver, CO, USA) was injected bilaterally at 5.5 mm caudal to the bregma, 1.2 mm lateral to the midline, and 4.5 mm in depth from the skull surface. To count the RGCs, isolated retinas were fixed in 4% paraformaldehyde and then mounted on a glass slide with the RGC layers facing up. FluoroGold-positive RGCs were identified under a fluorescence microscope using an ultraviolet light filter set (377/407 nm). RGCs were counted in 12 areas of 0.072 mm^2^ each (three areas per retinal quadrant) by an investigator blinded to the treatment conditions [4].

### 2.8. Electron Microscopy

The neuronal tissues were fixed in 10% glutaraldehyde for examination under a transmission electron microscope. Processing for electron microscopy was performed as described previously [14–16, 20, 21]. Images were captured in different regions of the optic nerve and lumbar spinal cord.

### 2.9. Western Blotting

At 3 days, 1 week and 8 weeks postimmunization (pi; *n* = 4/time point/group), whole-cell lysates were prepared from the lumbar spinal cord segments and brain tissue. The procedures used for Western blotting were as described previously [14–16, 20, 21]. As a negative control, the primary antibody was omitted.

### 2.10. Statistical Analysis

Data were analyzed using SPSS version 13.0. Values are presented as the mean ± standard deviation (SD). Comparisons between two groups were performed using unpaired Student's *t*-test. *P* < 0.05 was considered statistically significant. The statistical graphs were created with GraphPad Prism version 4.0 (GraphPad Prism Software Inc., San Diego, CA, USA).

## 3. Results

### 3.1. C + A Treatment Reduced the Disease Severity in NMO Rats

**Figure 1:**
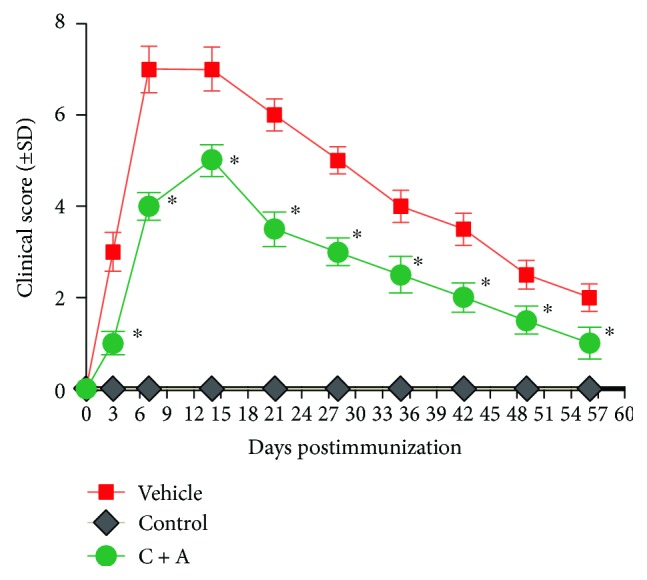
Combination treatment of C16 and Ang1 improves the clinical progression of NMO, as measured by disease scoring (*n* = 10 in each group). ^∗^ *P* < 0.01 versus the vehicle-treated group at the same time point. The onset of NMO symptoms in the vehicle group was at 3 days pi, and the symptoms peaked at 1 week pi and remained until 2 weeks pi. The clinical scores of the vehicle-treated rats started to decrease afterwards, and a limp tail remained at 8 weeks pi. However, animals treated with C + A displayed clinical signs of a delayed peak stage and a reduced severity at 2 weeks pi (with mild paraparesis of the hindlimb, clinical score = 5). Moreover, the clinical scores were evidently lower in the C + A-treated group than in the vehicle-treated group at each time point.

Functional scoring demonstrated that the onset of disease symptoms (absent righting, score = 3) was observed in the vehicle-treated rats at 3 days pi, and the symptoms peaked at 1 week pi (severe paraparesis, score = 7, Figure 1(a)) and remained until 2 weeks pi. The clinical scores of the vehicle-treated rats started to decrease afterwards, and a limp tail remained at 8 weeks pi. However, animals treated with C + A displayed clinical signs of a delayed peak stage and a reduced severity at 2 weeks pi (with mild paraparesis of the hindlimb, clinical score = 5). In addition, the clinical scores were significantly lower in the C + A-treated group than in the vehicle-treated group (Figure 1). After the peak stage, the clinical symptoms of the C + A-treated rats gradually returned to the level of a reduced tail tone (score = 1, Figure 1).

### 3.2. C + A Treatment Reversed Electrophysiological Dysfunction in NMO Rats

NMO induction increased the latency to waveform initiation and decreased the peak amplitude for both the cortical somatosensory-evoked potential (Table 1) and the motor-evoked potential (Table 1) recordings. However, C + A treatment significantly reduced the disease-associated delays in latency, which are related to the speed of conduction, and reversed the decrease in amplitude, which is related to the number of surviving fibers (Table 1, Supplementary Figure 1).

### 3.3. C + A Treatment Attenuated Parenchymal Infiltration of Inflammatory Cells in the CNS of NMO Rats, Recovered APQ4 Expression, and Increased GFAP Expression

**Figure 2:**
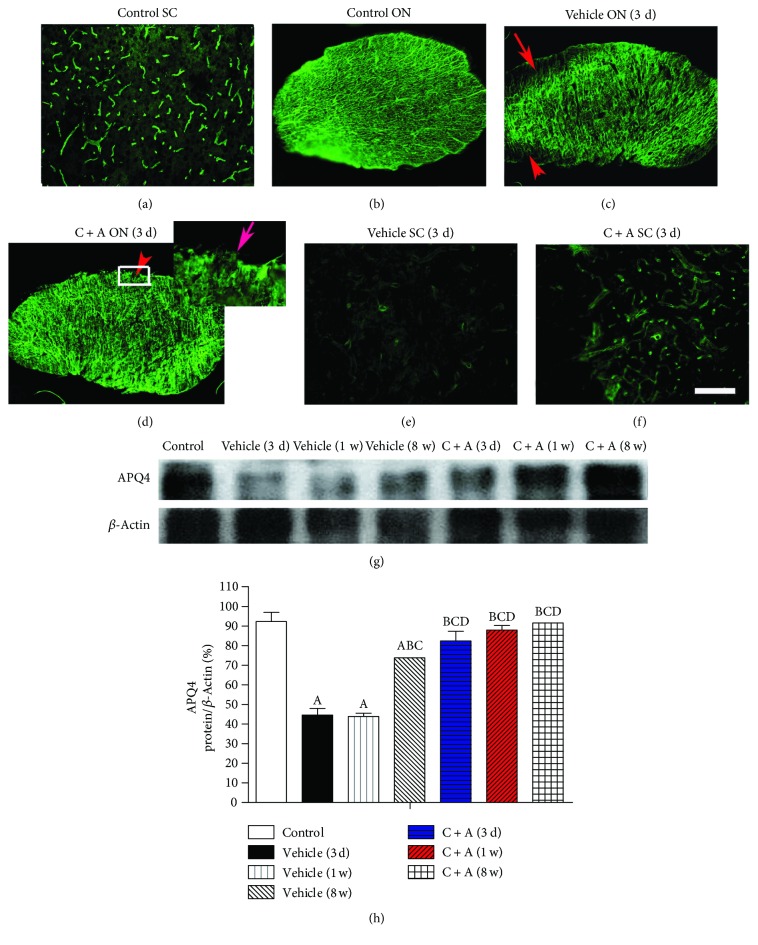
C + A treatment reversed the loss of APQ4 within the optic nerve and spinal cord. Expression of AQP4 was determined by immunostaining (green). Scale bar = 100 *μ*m. (a–f) Transverse sections through the spinal cord (SC) and optic nerve (ON). AQP4 is normally expressed on the plasma membranes of astrocytes closely apposing the basal membranes of blood vessels, both in the spinal cord (a) and in the optic nerves (b). AQP4 loss is visible in the optic nerves (arrow in c) and spinal cord (e) after NMO-IgG and human complement were intraventricularly/intrathecally infused for 3 days and optic nerve injections, while the combination treatment of C + A obviously reduced the APQ4 loss area (d, f). The arrow at high magnification of the square shows an APQ4 loss area in the optic nerve of the C + A-treated group, which was evidently smaller than in the vehicle-treated group. C + A treatment reversed the loss of APQ4 shown by Western blot (g, h). ^A^ *P* < 0.05 versus the control group. ^B^ *P* < 0.05 versus the vehicle-treated NMO rats at 3 days pi. ^C^ *P* < 0.05 versus the vehicle-treated NMO rats at 1 week pi. ^D^ *P* < 0.05 versus the vehicle-treated NMO rats at 8 weeks pi.

**Figure 3:**
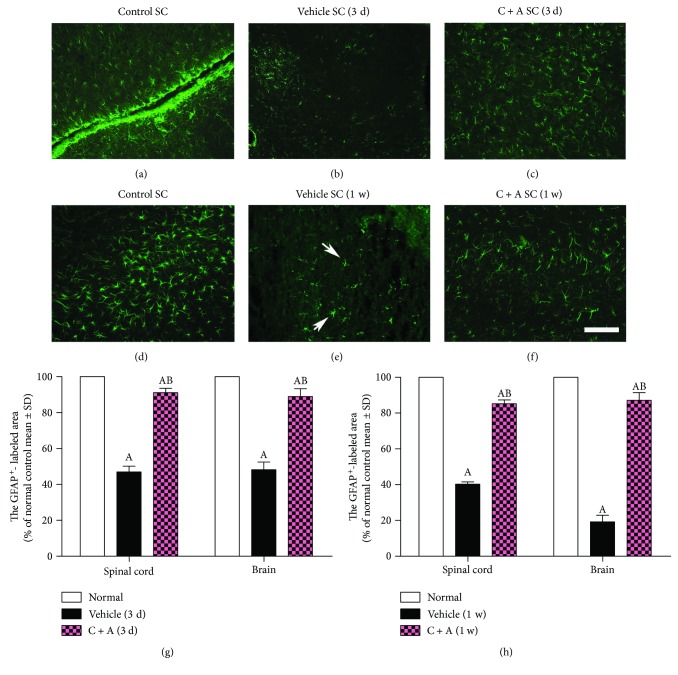
A visible GFAP loss was well-demonstrated in the spinal cord both at onset (3 days) and the peak stage (1 week) of control rats (b, e), and this loss was significantly reversed by C + A treatment (c, f). GFAP immunofluorescence; scale bar = 100 *μ*m. Transverse sections through the lumbar spinal cord white matter. The qualification of GFAP^+^ cells (g, h). ^A^ *P* < 0.05 versus the control group. ^B^ *P* < 0.05 versus the vehicle-treated NMO rats.

Expression of the AQP4, as determined by Western blot and IHC (Figure 2), and the GFAP, as determined by IHC (Figure 3), was significantly reduced in the optic nerves, spinal cord, and brain tissue of the vehicle-treated NMO rats. However, the reduction of AQP4 and GFAP expression was reversed by C + A treatment (Figures 2 and 3).

**Figure 4:**
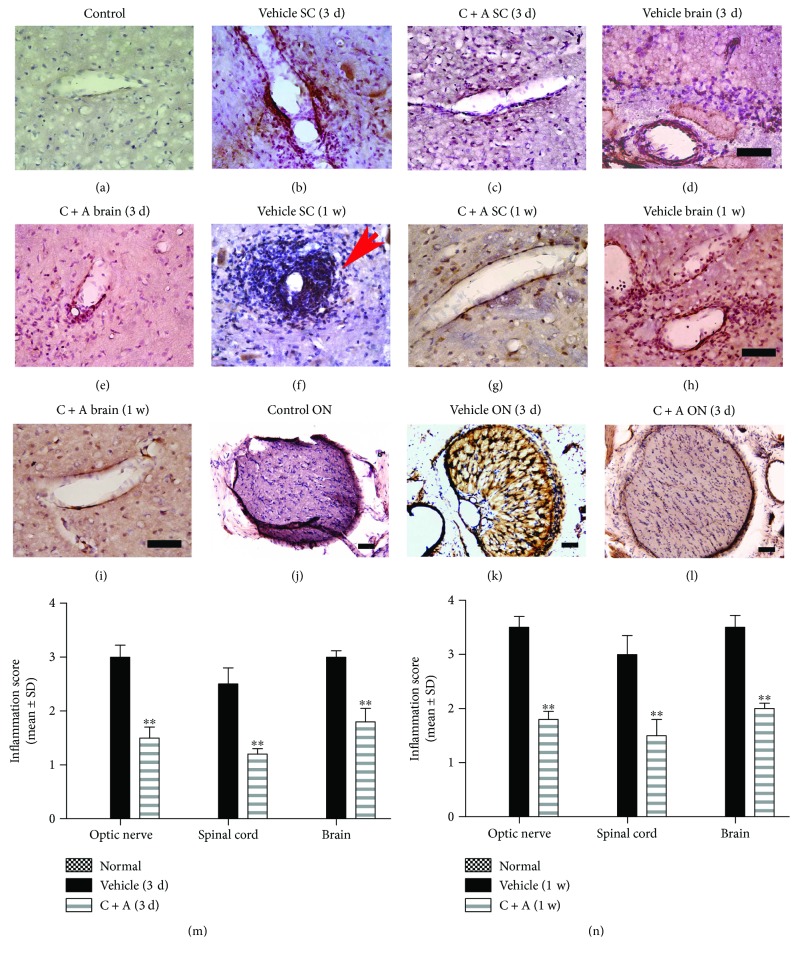
Infiltration of inflammatory CD45^+^ cells was observed in the parenchyma of the spinal cord (SC), brain, and optic nerve (ON) in the vehicle-treated NMO rats. The red arrow in (f) indicates infiltrated leukocytes surrounding blood vessels as “perivascular cuffing.” The C + A treatment evidently alleviated this condition. Immunostaining of CD45 and counterstaining with hematoxylin; scale bar = 100 *μ*m. Transverse sections through the brain (near the ventricle), lumbar spinal cord, and optic nerve. (m, n) C + A treatment attenuated CNS inflammation at 1 (m) and 8 (n) weeks pi, as shown by inflammation scoring. ^∗∗^ *P* < 0.01 versus the vehicle-treated NMO rats.

Perivascular and parenchymal infiltration of inflammatory CD45^+^ cells was significantly decreased in the optic nerves, spinal cord, and brain tissue in the C + A-treated NMO rats, compared to the vehicle-treated group (Figures 4(b)–4(l)). Consistently, the inflammatory score of the C + A-treated rats was significantly lower than that of the vehicle-treated NMO group at both 3 days and 1 week pi (Figures 4(m) and 4(n)).

### 3.4. C + A Treatment Reduced Demyelination and Axonal Loss in the CNS following NMO Induction

**Figure 5:**
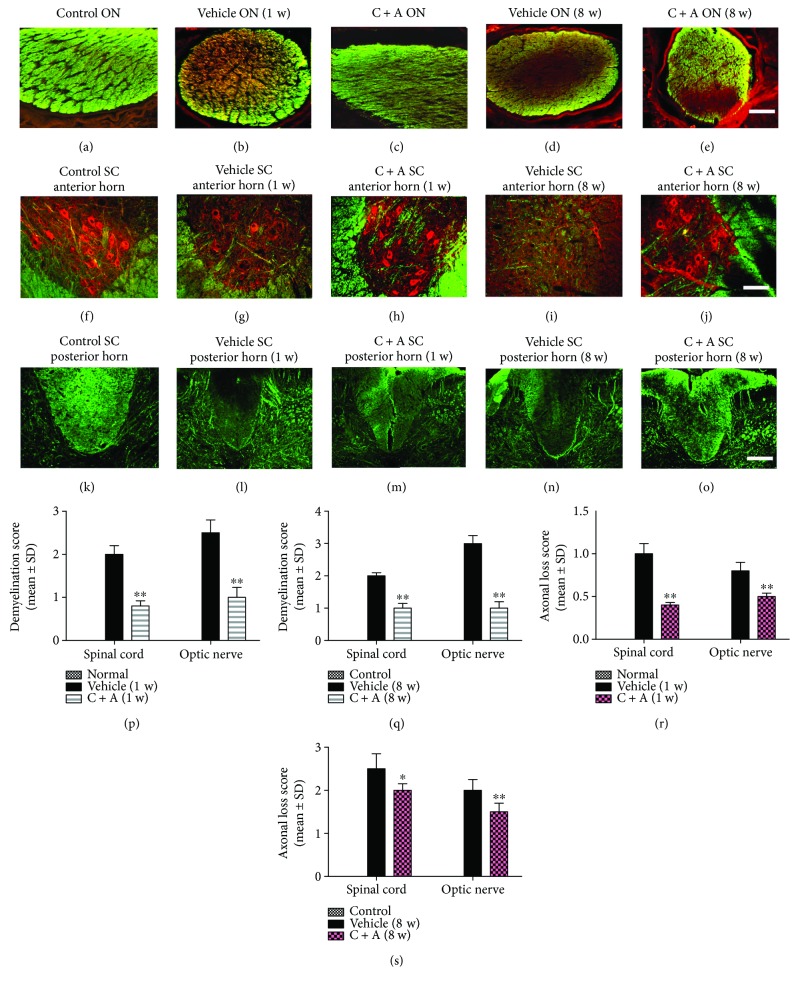
The vehicle-treated NMO rats demonstrated visible demyelination (shown by MBP immunofluorescence, green) in the optic nerve (a–e) and spinal cord (f–j, anterior horn; k–o, posterior fasciculus). Meanwhile, the loss of neurofilaments (shown by NF-M immunofluorescence, red) was also detected in neurons of the spinal cord anterior horn, especially at 8 weeks pi, the late stage of clinical progression (g, i). However, C + A treatment could reduce demyelination and axonal loss in the CNS following NMO induction. Scale bar = 100 *μ*m. (a–e) Transverse sections through the optic nerve. (f–j) Transverse sections through the anterior horn of the lumbar spinal cord. (k–o) Transverse sections through the posterior fasciculus of the lumbar spinal cord. (p–q) Demyelination was determined by demyelination scoring. C + A-treated rats exhibited notably less demyelination at both 1 (p) and 8 (q) weeks pi. ^∗∗^ *P* < 0.01 versus the vehicle-treated NMO rats. (r, s) C + A treatment preserved more axons in the CNS following NMO induction at both 1 (r) and 8 (s) weeks pi, which was demonstrated by the estimated axonal loss score. ^∗∗^ *P* < 0.05 versus the vehicle-treated NMO rats. ^∗∗^ *P* < 0.01 versus the vehicle-treated NMO rats.

Western blot analysis (Supplementary Figures 2A and 2B) and immunostaining of MBP, a marker of axonal myelination (Figure 5), showed that the vehicle-treated NMO rats had visible demyelization at 1 week pi, the clinical peak stage of NMO, in the optic nerve and spinal cord (Figures 5(b), 5(g), and 5(l); Supplementary Figures 2A and 2B), compared to the control rats (Figures 5(a), 5(f), and 5(k); Supplementary Figures 2A and 2B), while the rats treated with C + A had remarkably reduced demyelination (Figures 5(c), 5(h), and 5(m); Supplementary Figures 2A and 2B). At 8 weeks pi, although the demyelization area of the C + A treatment group was also increased compared to at 1 week pi (Figures 5(e), 5(j), and 5(o); Supplementary Figures 2A and 2B), both MBP immunostaining and Western blotting exhibited that the C + A-treated groups achieved greater areas of myelination, compared with the vehicle-treated rats (Figures 5(d), 5(i), and 5(n); Supplementary Figures 2A and 2B), which was consistent with the notably reduced demyelization scores in the C + A-treated group at both 1 and 8 weeks pi (Figures 5(p) and 5(q)).

At the clinical peak stage of NMO in the vehicle-treated rats, Western blotting (Supplementary Figures 2C and 2D) and immunostaining for NF-M (Figures 5, double stained with MBP), a marker of neurofilaments, revealed that the axonal density in the CNS was reduced (Figures 5(b) and 5(g); Supplementary Figures 2C and 2D) when compared with the control group (Figures 5(a) and 5(f); Supplementary 2C and 2D). But the C + A-treated rats did not exhibit remarkable axonal loss at 1 week pi, compared to vehicle treatment (Figures 5(c) and 5(h); Supplementary Figures 2C and 2D). Vehicle-treated NMO rats displayed more severe axonal loss at 8 weeks pi than at 1 week pi (Figures 5(d) and 5(i); Supplementary Figures 2C and 2D). In contrast, greater numbers of axons with a relatively normal morphology were observed in the C + A-treated NMO rats (Figures 5(e) and 5(j); Supplementary Figures 2C and 2D). Scoring of axonal loss at 1 and 8 weeks pi (Figures 5(r) and 5(s)) also confirmed the therapeutic effects of C + A treatment on axonal protection.

**Figure 6:**
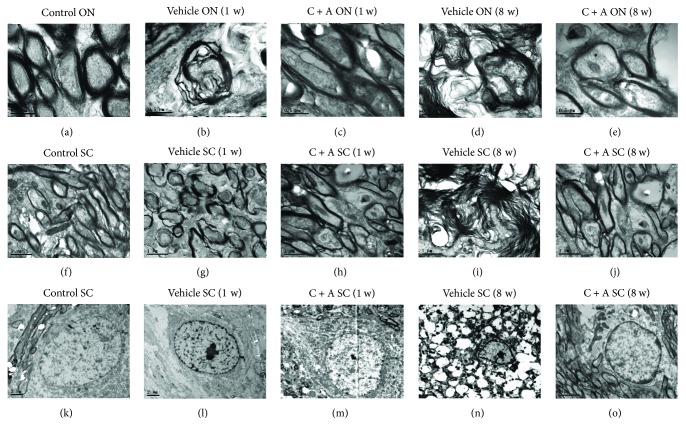
Electron micrograph demonstrating the prevention of perivascular edema, demyelination/axon loss, and neuronal apoptosis in the C + A-treated groups. (a, f, k) In control rats, the normal myelinated axons exhibited dark, ring-shaped myelin sheaths surrounding axons in both the optic nerve (a) and the white matter of the spinal cord (f); normal neuronal nuclei showed uncondensed chromatin (k). (b, g, l) Vehicle-treated NMO rats at 1 week pi. (b, g) Myelin sheath displaying splitting, vacuoles, and loose and fused changes, with atrophied axons (white arrow in b). (l) Apoptotic neuron with a shrunken nucleus and condensed and fragmented and margination of nuclear chromatin. (c, h, m) Meanwhile, in the C + A-treated rats, myelin sheath splitting and axonal loss were reduced. (d, i, n) At 8 weeks pi, more myelin sheaths were undergoing vesicular disintegration and demyelination, both in the optic nerve (d) and in the white matter of the spinal cord (i). Very severe tissue edema and apoptotic neurons with shrunken nuclei were found (n). In contrast, in the C + A-treated rats, more myelin sheaths surrounding intact axons were observed (e, j), while the morphology of the nucleus was relatively normal (o). (a–e) Scale bar = 0.5 *μ*m. (f–j) Scale bar = 1 *μ*m. (k–o) Scale bar = 2 *μ*m. (a–e) Transverse sections through the optic nerve. (f–o) Transverse sections through the anterior horn of the lumbar spinal cord.

Transmission electron microscopy showed that the non-NMO rats lacked edema around the blood vessels and appeared normal around the myelin sheath, axons, and cell nuclei (Figures 6(a), 6(f), and 6(k)). However, in the vehicle-treated NMO rats, the myelin sheath displayed splitting and vacuolar changes (Figures 6(b) and 6(g)), and neurons showed signs of apoptosis (Figure 6(l)) at 1 week pi; while in the C + A-treated NMO rats, fewer vacuolated myelin sheaths (Figures 6(c) and 6(h)) were observed in both the optic nerve and white matter of the spinal cord. At 8 weeks pi, notable demyelination and severe edema (Figures 6(d), 6(i), and 6(n)) were detected in the vehicle-treated group, but myelinated fibers were relatively intact in the C + A-treated rats (Figures 6(e) and 6(j)). Furthermore, their neighboring nuclei showed a normal ultrastructure (Figure 6(o)).

### 3.5. C + A Treatment Reduced Neuronal Loss in NMO Rats

**Figure 7:**
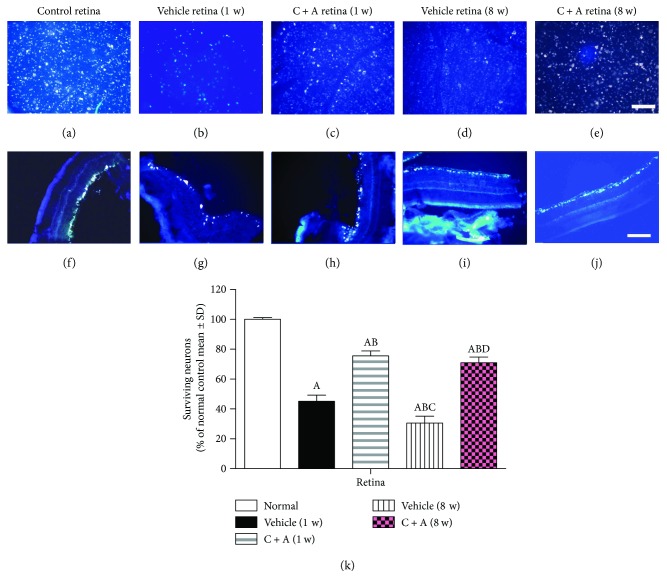
NMO-IgG infusion decreased the number of retinal ganglion cells labeled by the dye FluoroGold, as shown by the retinal stretched preparation (b, d) and coronal sections through the eyeball (g, i); however, the C + A treatment could remarkably reverse this phenomenon (c, h, e, j). Bar = 100 *μ*m. (k) The counting of surviving retinal ganglion cells in different treatment groups (% of control group). ^a^ *P* < 0.05 versus the control group. ^b^ *P* < 0.05 versus the vehicle-treated NMO rats at 1 week pi. ^c^ *P* < 0.05 versus the C + A-treated NMO rats at 1 week pi. ^d^ *P* < 0.05 versus the vehicle-treated NMO rats at 8 weeks pi.

Figures 7(b) and 7(g) show that the number of RGCs in the retinas of vehicle-treated NMO rats was significantly reduced at 1 week pi and was more remarkable at 8 weeks pi (Figures 7(d) and 7(i)). However, in the C + A treatment group, 70% of the RGCs survived, compared to the control group (Figure 7(k)).

**Figure 8:**
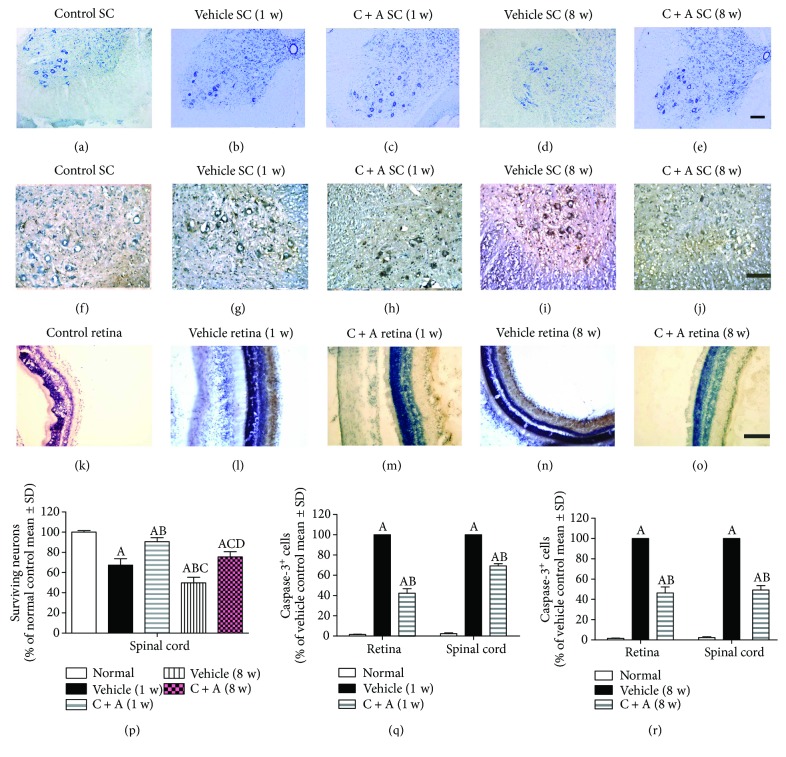
(a–e) Treatment with C + A reduced neuronal loss in the spinal cord and brain as verified by Nissl staining. Transverse sections through the anterior horn of the lumbar spinal cord; scale bar = 100 *μ*m. (f–o) In the vehicle-treated NMO rats, the expression of active caspase-3 was markedly increased (g, l, i, n). This phenomenon could be reversed by C + A treatment (h, m), which was the most clear at 8 weeks pi (j, o), as shown by the determination of the immunostained caspase-3-positive cells and counterstaining with hematoxylin. (f–j) Transverse sections through the anterior horn of the lumbar spinal cord. (k–o) Coronal sections through the eyeball. Scale bar = 100 *μ*m. (p) Surviving neural cells calculated in different groups at 1 and 8 weeks pi following Nissl staining (each group is presented as a percentage of the control group). ^a^ *P* < 0.05 versus the control group. ^b^ *P* < 0.05 versus the vehicle-treated NMO rats at 1 week pi. ^c^ *P* < 0.05 versus the C + A-treated NMO rats at 1 week pi. ^d^ *P* < 0.05 versus the vehicle-treated NMO rats at 8 weeks pi. (q, r) The qualification of caspase-3^+^ cells at 1 (q) and 8 (r) weeks pi. ^a^ *P* < 0.05 versus the control group. ^b^ *P* < 0.05 versus the vehicle-treated NMO rats.

In the vehicle-treated NMO group, significant neuronal loss (Figures 8(b) and 8(d)) was also observed in the CNS of the vehicle-treated NMO rats, especially at 8 weeks pi (Figure 8(d)) when compared to control rats (Figure 8(a)), as shown by Nissl staining (Figures 8(a)–8(e)). Nevertheless, in the C + A-treated NMO rats, more neurons were present in the anterior horn of the spinal cord (Figures 8(c) and 8(e)). The number of neurons in the CNS of C + A-treated rats was significantly higher than that in the vehicle-treated groups (Figure 8(p); *P* < 0.05).

The expression of activated caspase-3, responsible for the execution of apoptosis, was upregulated in neurons of the spinal cord anterior horn and retina in the vehicle-treated NMO rats, compared with the control group (Figures 8(g), 8(i), 8(l), and 8(n)). This effect was reversed by C + A (Figures 8(h), 8(j), 8(m), and 8(o)) treatment at both 1 (Figure 8(q)) and 8 weeks pi (Figure 8(r)).

## 4. Discussion

Asavapanumas et al. employed the recombinant monoclonal NMO antibody rAb-53 (referred to as NMO-IgG), which was derived from a clonally expanded plasma blast population from cerebrospinal fluid of an NMO patient, and human complement to produce a mouse model of NMO [4]. Their results showed that coinjection of NMO-IgG and human complement near the optic chiasma or at the L5–L6 spinal cord of mice can produce NMO-like optic nerve pathology or longitudinally extensive white-matter lesions in the lumbar spinal cord [4]. Meanwhile, Matsumoto et al. collected AQP4 antibody from Japanese NMO patients with NMO spectrum disorders. An intravitreal injection of 1 *μ*g of NMO-IgG and 0.5 *μ*L of human complement was used to induce a rodent NMO model in Sprague-Dawley rats [5]. In addition, Ratelade et al. injected 2 *μ*g of AQP4-IgG and 3 *μ*L of 20% human complement in 8 *μ*L of PBS intracerebrally into the brain of mice [1], and Zhang and Verkman induced demyelinating NMO lesions by continuous and chronic intracerebral infusion of NMO-IgG and complement in the lateral brain of mice [6]. Moreover, intracerebral injection of NMO immunoglobulin G and human complement produced extensive inflammatory cell infiltration, extensive demyelination, loss of aquaporin-4 expression, loss of reactive astrocytes, and neuronal cell death [22]. Ratelade et al. found that in the absence of complement, AQP4-IgG and natural killer cells can produce NMO-like lesions with loss of GFAP and AQP4, but no loss of myelin [1]. Furthermore, Grunewald et al. [7] attempted the chronic intrathecal infusion of purified IgG fractions from NMO patients into rat cerebrospinal fluid and produced the NMO rodent models without the use of human complement. In our studies, we took advantage of these previous studies to produce our NMO animal model. The anti-AQP4 antibodies derived from the serum of patients and human complement were used for generating a NMO rodent model that was based on the anti-AQP4-mediated astrocyte damage in Lewis rats, which are prone to develop spontaneous autoimmune neuroinflammatory diseases. We established the rat NMO model successfully by intraventricular and intrathecal injection into the cerebrospinal fluid. This model reproduced multiple pathological characteristics and symptoms of NMO [1, 4–8]. Furthermore, based on multiple lines of evidence, including relief of NMO symptoms, suppression of inflammation and demyelination, as well as reduction of axonal loss and neuronal apoptosis, we demonstrated that the combination therapy of C16 plus Ang1 was an effective treatment for NMO in this model.

Most patients are seropositive for NMO-IgG, which specifically interacts with AQP4 [17, 23]. AQP4 is a bidirectional water channel expressed on the plasma membranes of astrocytes in the brain and spinal cord, retinal Müller cells, and optic nerves, with AQP4 on the membranes closely apposing endothelial cell basal membranes [24]. The active interactions between astrocytes and endothelial cells are critical in maintaining the BBB to limit the access of immune system effectors [25, 26]. Analyses of AQP4-deficient mice have revealed its involvement in cerebral water and ion homeostasis regulation, astrocyte migration, and neural signal transduction, thus demonstrating its role for the maintenance of BBB integrity [26].

Previous studies have clearly demonstrated that NMO-IgG is pathogenic in inducing or exacerbating the disease [1]. It is believed that NMO-IgG can pass the BBB to attack astrocytes in the CNS by binding to AQP4, leading to astrocyte damage due to complement-dependent cytotoxicity. That is the acute lesion caused by the passive transfer of NMO-IgG and human complement, and in our study, the evident reduction of APQ4 and astrocyte marker expression happened at 3 days pi, the onset stage of disease, immediately following the NMO-IgG and complement infusion, and remained until 1 week pi, the peak stage of NMO.

Following the acute lesion, the secondary lesions lead to further pathological changes. It has been shown that the interaction between NMO-IgG and cells of the neurovascular unit (astrocytes and the brain endothelium) alters BBB permeability and granulocyte recruitment [27, 28]. The damage of astrocytes closely apposing endothelial basal membranes can induce extensive inflammatory cell infiltration [4]. Increased granulocyte recruitment also increases the release of chemotactic factors, which can stimulate and attract neutrophils, further resulting in massive perivascular recruitment of inflammatory cells into the brain, optic nerve, and spinal cord. Consistently, our pathological studies showed widespread perivascular and parenchymal infiltration of CD45^+^ (leukocyte common antigen) leukocytes in the CNS of vehicle-treated NMO rats. Since extravasated inflammatory cells can activate a series of noxious factors and destroy the microenvironment in the CNS, they ultimately cause secondary injury. Because the oligodendrocytes are highly vulnerable to the inflammatory milieu, inflammation may lead to demyelination, which is probably secondary to oligodendrocyte apoptosis due to loss of trophic support from astrocytes. Extensive demyelination leads to the denudement of axons and destruction of normal axons. Ultimately, extensive axonal injury causes clinical symptoms such as sensory/motor impairment and paralysis [22, 29]. In electrophysiological tests of cortical somatosensory-evoked potentials and motor-evoked potential, a delayed latency is more indicative of focal demyelination, and a decrease in amplitude indicates the extent of axonal damage. In our C + A-treated rats, the alleviated infiltration of inflammatory cells and the improved microenvironment could lessen oligodendrocyte and neuronal apoptosis, decrease axon demyelination, and increase the number of surviving neurons (with intact axons), finally contributing to the amelioration of disease progression and severity.

The integrin *α*v*β*3 is critical for leukocyte-endothelium interactions and transmigration of leukocytes across the endothelium [30]. C16, as a functional laminin domain, specifically binds to integrin; therefore, it competitively reduces leukocyte-endothelium interactions and blocks the transmigration of leukocytes across the endothelium [13, 31, 32]. Moreover, the integrin *α*v*β*3 is also an important receptor that regulates macrophage differentiation and responses to external signals. Furthermore, *α*v*β*3 activation can maintain chronic inflammatory processes in pathological conditions [30]. Thus, the blockage of *α*v*β*3 may also directly inhibit macrophage-related inflammation.

A case series study of five patients by Kleiter et al. has shown that Natalizumab is not beneficial for NMO, although it is an effective treatment for relapsing-remitting multiple sclerosis (MS) [33]. This result is not likely to be conclusive, since the number of cases was so few. Natalizumab exerts its efficacy by blocking the *α*4 integrin-mediated adhesion of immune cells to the BBB [34]. In this respect, Natalizumab is similar to C16 (but not similar to Ang1). However, C16 interacts with different integrins, *ανβ*3 and *α*5*β*1. Moreover, C16 also works as an *α*ν*β*3 agonist and promotes angiogenesis [31, 34, 35]. Thus, the different mechanisms of Natalizumab and C16 plus Ang1 may produce different effects in the treatment of NMO.

Ang1, as an antileakage factor, modulates the BBB during acute injury and inflammation, can reduce vascular permeability, and promotes vessel survival [16, 36]. In our study, Ang1 infusion into the blood vessels may reduce the amount of NMO-IgG diffused into the CNS and alleviate its attack on APQ4 on astrocytes, whose plasma membranes surround blood vessels. Previous studies also have revealed that Ang1 can reduce the size of endothelial gaps by inducing the expression of adhesive PECAM-1 and the tight junction proteins occludin and ZO-2, further reinforcing vessel integrity by inhibiting the transcription of genes associated with vessel destabilization and remodeling [28].

In addition, by securing paracellular junctions, Ang1 also effectively limits the progression of the inflammatory response by inhibiting the expression of the inflammatory cytokine nuclear factor-*κ*B and the adhesion molecules intercellular adhesion molecule 1, vascular cell adhesion molecule 1, and E-selectin, which are required for the migration of inflammatory cells [28]. Our data show that the severe edema in the CNS of vehicle-treated rats was significantly alleviated by C + A application, which was in concert with the above properties of Ang1.

Studies in an MS model have shown that C16 effectively suppresses inflammatory cell infiltration [15, 20, 21] and that Ang1 effectively reduces vascular leakage [16]. Treatment with a combination of Ang1 and C16 is superior to treatment with either Ang1 or C16 alone [14]. Therefore, we adopted the combination of C + A to target inflammatory cell transmigration and vascular permeability to test its therapeutic effects on NMO, with the ratio and dosage based on our previous studies [12, 14, 36]. Our data demonstrated that the widespread perivascular and parenchymal infiltration of leukocytes in the CNS of NMO rats was significantly suppressed by intravenous injection of the combination of C + A.

## 5. Conclusions

Although the pathogenesis of MS and NMO as well as the expression patterns of APQ4 and GFAP in these two diseases is quite different [2, 37], these two distinct diseases share common processes, including BBB disruption, activation of chemotactic factors, and extensive infiltration of inflammatory cells. Besides, other neuronal disorders such as cerebral ischemia, traumatic brain injury, neuropathic pain, Alzheimer's disease, migraine, autism, and depression are also characterized by the appearance of neuroinflammation, which contribute to the death of motor neurons [38]. The neuroinflammatory processes may represent a therapeutic target to alleviate neurodegeneration. Our results suggest that even though the pathogenesis causing the inflammatory milieu of different diseases is different, targeting the key points of inflammatory cell transmigration and improving vascularity will achieve therapeutic effects in ameliorating the inflammatory milieu of inflammation-related CNS diseases, which usually involve BBB disruption and extravasation of inflammatory cells. This finding broadens our thinking regarding the treatments of inflammation-related CNS diseases.

## Figures and Tables

**(a):** 

Group	N	P	Wave amplitude (*μ*V, mean ± SD)
1-week c-SEP	Latency (ms); *n* = 8		
Control	13.95 ± 0.31^∗^	19.68 ± 0.17^∗∗^	12.39 ± 0.68^∗∗^
Vehicle	22.31 ± 0.32	28.99 ± 0.44	3.48 ± 0.28
C16 + Ang1	16.48 ± 0.18^∗^	20.21 ± 0.19^∗∗^	10.89 ± 1.32^∗∗^
8-week c-SEP	Latency (ms); *n* = 8		
Control	12.88 ± 0.34^∗^	17.69 ± 0.25^∗∗^	14.08 ± 1.24^∗∗^
Vehicle	29.3 ± 1.22	37.68 ± 0.65	2.55 ± 0.16
C16 + Ang1	15.67 ± 0.06^∗^	20.59 ± 0.1^∗∗^	14.89 ± 0.88^∗∗^

**(b):** 

Group	Latency (ms)	Wave amplitude (*μ*V, mean ± SD)
1-week MEP	*n* = 8	
Control	6.12 ± 0.24^∗^	10.42 ± 1.22^∗∗^
Vehicle	6.86 ± 0.64	0.12 ± 0.03
C16 + Ang1	5.78 ± 0.15^∗^	4.45 ± 0.18^∗∗^
8-week MEP	*n* = 8	
Control	5.76 ± 0.23^∗∗^	10.09 ± 0.61^∗∗^
Vehicle	6.57 ± 0.95	0.72 ± 0.02
C + A + R	5.29 ± 0.8^∗∗^	6.33 ± 0.35^∗∗^

^∗^ *P* < 0.05 versus the vehicle-treated rats. ^∗∗^ *P* < 0.01 versus the vehicle-treated rats. N: negative deflection; P: positive deflection; c-SEP: somatosensory-evoked potential; MEP: motor-evoked potential.

## Supplementary Materials

Supplementary Materials Supplementary Figure 1: (A-B) combination treatment of C16 + Ang1 reduced the clinical severity of NMO in rats at 1 (A) and 8 (B) weeks pi, as measured by determining the somatosensory-evoked potential (c-SEP) latencies and amplitudes, measured from peak to peak between negative deflection (N) and positive deflection (P). The amplitude of c-SEP was notably lower in the vehicle-treated EAE rats, and the latency was also significantly prolonged, while treatment with C + A effectively reversed these phenomena. (C-D) The motor-evoked potential (MEP) amplitude was also significantly lower in the vehicle-treated NMO rats, and the latency was also significantly prolonged when compared with normal rats. Similarly, these phenomena were reversed following C + A treatment. Supplementary Figure 2: C + A treatment significantly reversed the decrease of MBP at both 1 and 8 weeks pi (A-B) and NF (C-D) expression at 8 weeks pi in the CNS when compared with the vehicle-treated NMO rats. Moreover, it reduced the expression of the apoptosis-related enzyme caspase-3 (E-F), as shown by Western blotting. (a) *P* < 0.05 versus the normal control; (b) *P* < 0.05 versus the vehicle-treated NMO rats at 1 week pi; (c) *P* < 0.05 versus the C + A-treated NMO rats at 1 week pi; and (d) *P* < 0.05 versus the vehicle-treated NMO rats at 8 weeks pi.
