# Supplementary material for: Combination Treatment of C16 Peptide and Angiopoietin-1 Alleviates Neuromyelitis Optica in an Experimental Model
Source: Mediators Inflamm. 2018 Feb 18;2018:4187347. doi: 10.1155/2018/4187347 (PMC5835265; doi:10.1155/2018/4187347)
Supplement: Supplementary Materials — Supplementary Figure 1: (A-B) combination treatment of C16 + Ang1 reduced the clinical severity of NMO in rats at 1 (A) and 8 (B) weeks pi, as measured by determining the somatosensory-evoked potential (c-SEP) latencies and amplitudes, measured from peak to peak between negative deflection (N) and positive deflection (P). The amplitude of c-SEP was notably lower in the vehicle-treated EAE rats, and the latency was also significantly prolonged, while treatment with C + A effectively reversed these phenomena. (C-D) The motor-evoked potential (MEP) amplitude was also significantly lower in the vehicle-treated NMO rats, and the latency was also significantly prolonged when compared with normal rats. Similarly, these phenomena were reversed following C + A treatment. Supplementary Figure 2: C + A treatment significantly reversed the decrease of MBP at both 1 and 8 weeks pi (A-B) and NF (C-D) expression at 8 weeks pi in the CNS when compared with the vehicle-treated NMO rats. Moreover, it reduced the expression of the apoptosis-related enzyme caspase-3 (E-F), as shown by Western blotting. (a) P < 0.05 versus the normal control; (b) P < 0.05 versus the vehicle-treated NMO rats at 1 week pi; (c) P < 0.05 versus the C + A-treated NMO rats at 1 week pi; and (d) P < 0.05 versus the vehicle-treated NMO rats at 8 weeks pi. [file 4187347.f1.doc]

**Combination treatment of C16 peptide and Angiopoietin-1 Alleviates Neuromyelitis Optica (NMO) in an Experimental Model**

**Supplementary Figure Legends**

**Supplementary Figure 1.** (A–B) Combination treatment of C16+Ang1 reduced the clinical severity of NMO in rats at 1 (A) and 8 (B) weeks pi, as measured by determining the somatosensory-evoked potential (c-SEP) latencies and amplitudes, measured from peak to peak between negative deflection (N) and positive deflection (P). The amplitude of c-SEP was notably lower in the vehicle-treated EAE rats, and the latency was also significantly prolonged, while treatment with C+A effectively reversed these phenomena. (C–D) The motor-evoked potential (MEP) amplitude was also significantly lower in the vehicle-treated NMO rats, and the latency was also significantly prolonged when compared with normal rats. Similarly, these phenomena were reversed following C+A treatment.

**Supplementary Figure 2.** C+A treatment significantly reversed the decrease of MBP at both 1 and 8 weeks pi (A–B), and NF (C–D) expression at 8 weeks pi in the CNS when compared with the vehicle-treated NMO rats. Moreover, it reduced the expression of the apoptosis-related enzyme caspase-3 (E–F), as shown by western blotting. (a), P < 0.05 versus the normal control; (b), P < 0.05 versus the vehicle-treated NMO rats at 1 week pi; (c), P < 0.05 versus the C+A-treated NMO rats at 1 week pi; (d), P < 0.05 versus the vehicle-treated NMO rats at 8 weeks pi.

**
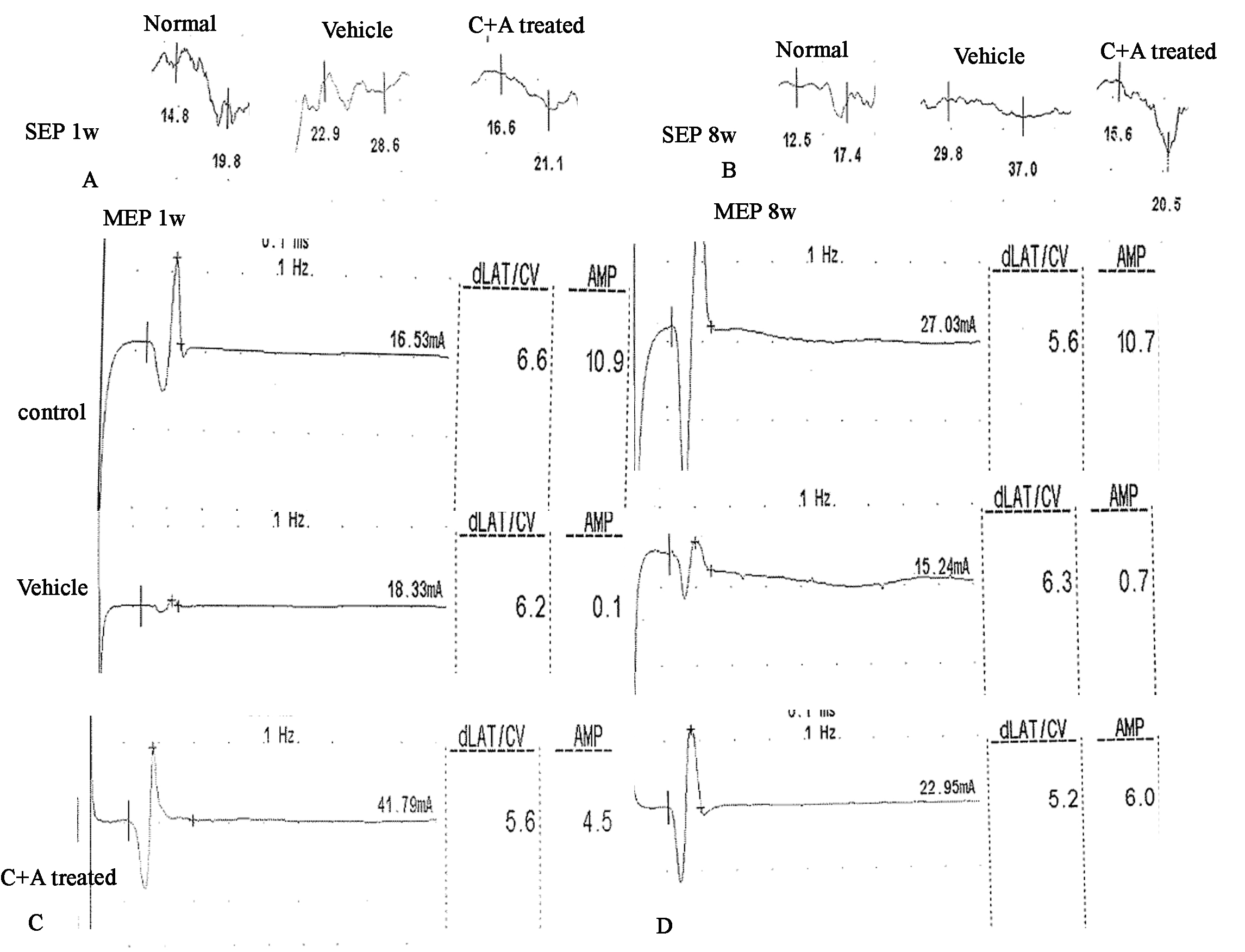
**

**Supplementary Figure 1**

**
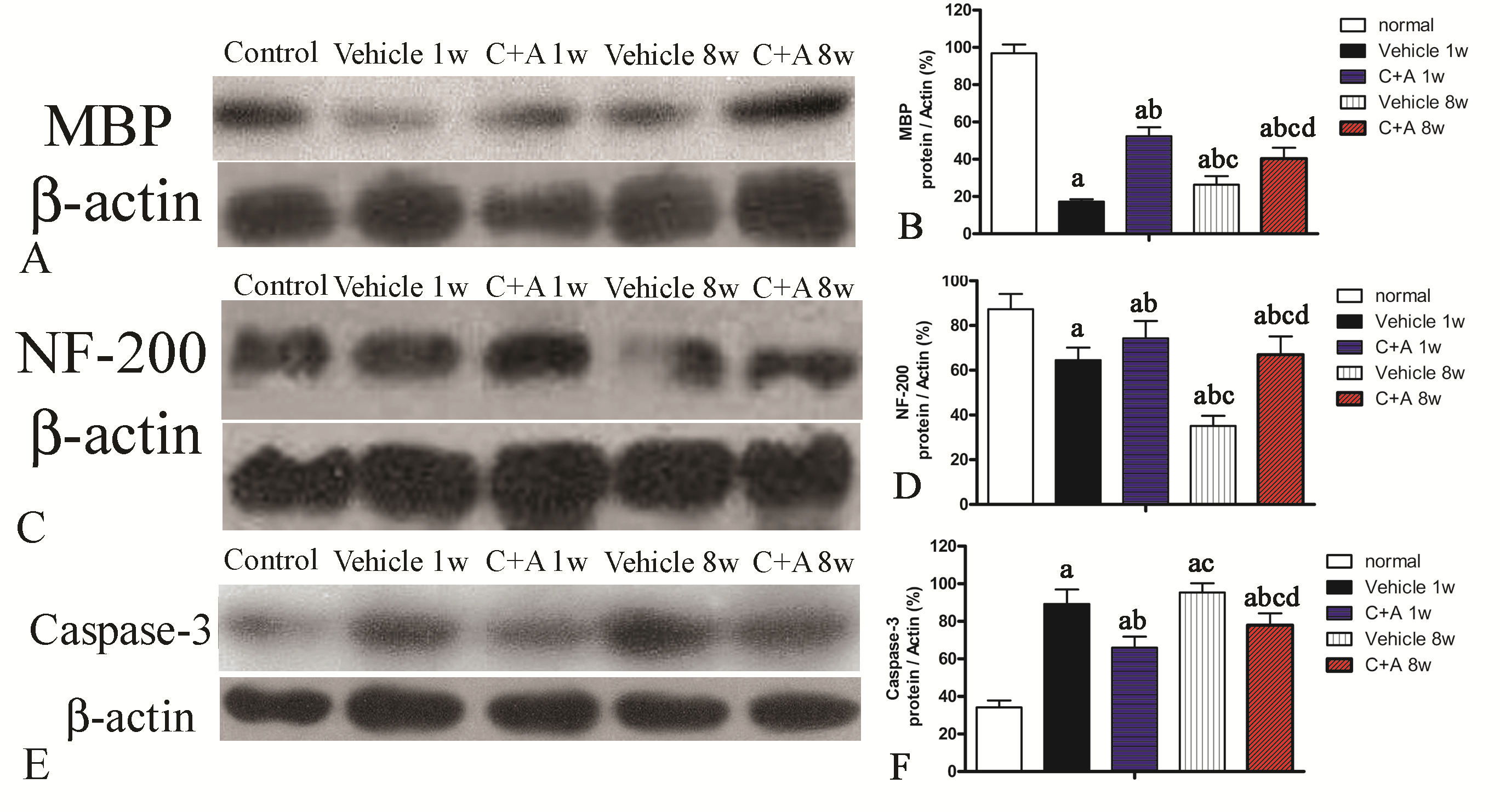
**

**Supplementary Figure 2**
